# Using CT texture analysis to differentiate between peripheral lung cancer and pulmonary inflammatory pseudotumor

**DOI:** 10.1186/s12880-020-00475-2

**Published:** 2020-07-06

**Authors:** Chenlu Liu, Changsheng Ma, Jinghao Duan, Qingtao Qiu, Yanluan Guo, Zhenhua Zhang, Yong Yin

**Affiliations:** 1 School of Nuclear Science and Technology, University of South China, Hengyang, 421001 China; 2 Department of Radiotherapy, Shandong Cancer Hospital and Institute, Shandong First Medical University and Shandong Academy of Medical Sciences, Jinan, 250117 Shandong Province China; 3 Department of Medical Imaging, Shandong Cancer Hospital and Institute, Shandong First Medical University and Shandong Academy of Medical Sciences, Jinan, 250117 Shandong Province China

**Keywords:** Radiomics features, Peripheral lung cancer, PET/CT, Pulmonary inflammatory pseudotumor

## Abstract

**Background:**

This study is to distinguish peripheral lung cancer and pulmonary inflammatory pseudotumor using CT-radiomics features extracted from PET/CT images.

**Methods:**

In this study, the standard 18F-fluorodeoxyglucose positron emission tomography/ computed tomography (18 F-FDG PET/CT) images of 21 patients with pulmonary inflammatory pseudotumor (PIPT) and 21 patients with peripheral lung cancer were retrospectively collected. The dataset was used to extract CT-radiomics features from regions of interest (ROI), The intra-class correlation coefficient (ICC) was used to screen the robust feature from all the radiomic features. Using, then, statistical methods to screen CT-radiomics features, which could distinguish peripheral lung cancer and PIPT. And the ability of radiomics features distinguished peripheral lung cancer and PIPT was estimated by receiver operating characteristic (ROC) curve and compared by the Delong test.

**Results:**

A total of 435 radiomics features were extracted, of which 361 features showed relatively good repeatability (ICC ≥ 0.6). 20 features showed the ability to distinguish peripheral lung cancer from PIPT. these features were seen in 14 of 330 Gray-Level Co-occurrence Matrix features, 1 of 49 Intensity Histogram features, 5 of 18 Shape features. The area under the curves (AUC) of these features were 0.731 ± 0.075, 0.717, 0.748 ± 0.038, respectively. The *P* values of statistical differences among ROC were 0.0499 (F9, F20), 0.0472 (F10, F11) and 0.0145 (F11, Mean4). The discrimination ability of forming new features (Parent Features) after averaging the features extracted at different angles and distances was moderate compared to the previous features (Child features).

**Conclusion:**

Radiomics features extracted from non-contrast CT based on PET/CT images can help distinguish peripheral lung cancer and PIPT.

## Background

Lung cancer is the world’s leading cause of cancer-related deaths and a highly malignant tumor [1, 2], According to the location of the tumor, lung cancer can be divided into peripheral lung cancer and central lung cancer, and more than 70% of all lung cancers are peripheral lung cancer [3]. lung cancer can be divided into adenocarcinoma, squamous cell carcinoma, small cell lung cancer and large cell lung cancer depending on the histological and cytological types of tumors. In recent years, with the improvement of the level of cancer diagnosis and treatment [4], the mortality rate of lung cancer has been on a downward trend, but lung cancer is still the leading cause of death [5]. About 70% of the patients with lung cancer are in the advanced stage at the time of initial diagnosis [6], and even some patients have already had metastasis, and if patients with lung cancer can be accurately diagnosed and undergo early surgery, the 10-year survival rate can reach 88% [7]. thus, the early diagnosis and timely treatment of patients with lung cancer are very important [8].

The good diagnostic effect of computed tomography (CT) has been achieved in the diagnosis of lung cancer. However, the traditional CT diagnosis of lung lesions is mainly based on morphology, which has certain limitations in the differential diagnosis of benign and malignant tumors [9, 10].

In recent years, 18F-fluorodeoxyglucose positron emission tomography/computed tomography (18F-FDG PET/CT) has achieved a great effect in the examination of chest lesions [11], and has been widely used in clinic [8]. Some reports have shown that PET/CT is more effective than conventional CT in identifying benign and malignant chest lesions [12, 13]. However, FDG is not a drug specifically ingested by cancer [13]. In most cases, the cumulative increase in metabolic activity of inflammatory cells will lead to an increase in FDG uptake, which is false positive on PET-CT. To some extent, these lesions can also mimic the biological behavior of malignant tumors. Even if experienced nuclear medicine physicians manage to distinguish between benign and malignant FDG uptake, The result is still far from satisfactory. This is a great challenge for clinicians and nuclear medicine doctors, such as the differentiation of pulmonary inflammatory pseudotumor (PIPT) and peripheral lung cancer [14, 15].

The CT signs of PIPT are usually complex, variable and unspecific, and showing round or oval, well-defined solitary peripheral nodules or masses [16–19], accounting for about 1% of adult lung tumors [20]. It is a rare disease characterized by pulmonary neoplastic hyperplasia of inflammatory cells, which is very similar to malignant tumors of the lung [21, 22]. PIPT occasionally has a process of invasive proliferation and usually shows false positive on PET/CT [18]. Due to the overlap between PIPT and peripheral lung cancer in histopathology, biological behavior, clinical manifestations and imaging features [20, 23], the clear diagnosis and differentiation often can’t be made by clinicians and nuclear medicine physicians.

Radiomics widely used in modern medical research, due to radiomics can quantitative tumor heterogeneity with the advantages of repeatable, non-invasive, free from time and space constraints and low-cost [24, 25]. In recent years, radiomics plays a more and more important role in the qualitative diagnosis of tumors, the differentiation of benign from malignant tumors and the evaluation of prognosis of radiotherapy response. The purpose of this study is to explore whether PIPT and peripheral lung cancer can be distinguished by radiological analysis.

## Methods

### Radiomics workflow

**Fig. 1:**
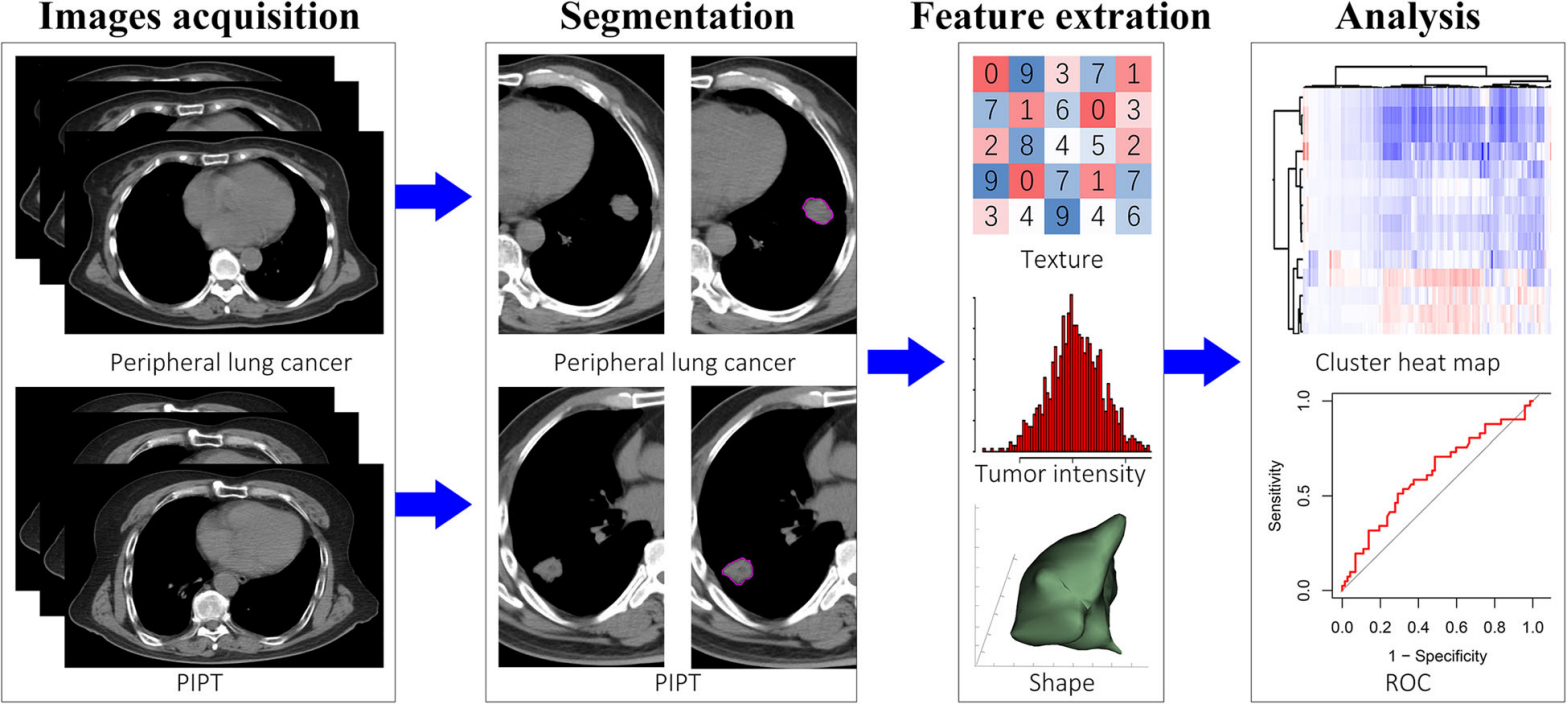
The workflow of this study

The raidomics flow of this study included: (1) images acquisition, (2) image segmentation, (3) feasure extraction, (4) data analysis. All the steps are shown in Fig. 1.

### Patients

This study was approved by the Ethics Review Committee of Shandong Cancer Hospital and informed consent was waived. The collection of patient materials in this study was carried out in two steps. The first step enrolled PIPT patients who had undergone 18F-FDG PET/CT scans in our hospital from May 2015 to October 2019, and their pathological diagnosis was available. In the second step, the images of the patients with peripheral lung cancer were included randomly. The criteria for patient inclusion were as follows: (I) the patient underwent PET/CT examination for the first time; (II) the patient had no history of diabetes; (III) the maximum diameter of the lesion was not less than 1.5 cm; (IV) the patient had complete clinical materials and pathological reports; (V) standardized uptake value of lesions ≥ 2.5.

### Patient images acquisition

The 18F-FDG PET/CT images of all patients were obtained by the same hybrid PEC/CT scanner (Philips Healthcare, Cleveland, OH). The patient fasted for more than 6 h, and the blood glucose level of all patients was normal before the scan. PET/CT scans were completed 60 min after intravenous injection of 18F-FDG at 4.4 MBq/kg. After attenuation correction and iterative reconstruction, the PET images were used for multi-plane and multi-image imaging, and the images were fused with the CT images. The initial CT image acquisition was conducted with slice thickness 2 mm (120KVp, 206 mA), and reconstructed to a 512 × 512 matrix (voxels size, 1.17 × 1.17 × 5 mm^3^). Then, the obtained image data can be viewed in coronal, sagittal, and axial planes using image workstation.

### Region of interests (ROI) segmentation

The total process of image segmentation was completed by three experienced doctors. All the image data were imported into the MIM software (Cleveland, OH), and the total process of image segmentation was completed by three experienced radiologists. In the first group of experiments, ROI was manually delineated by a radiologist with 15 years of experience on the MIM software and supervised by another nuclear medicine physician with 20 years of experience, a clinical chest doctor with 20 years of experience to examine and correct all the delineated images. In the second group of experiments, ROI was manually delineated by a nuclear medicine physician with 20 years of experience, The other two doctors checked all images and discussed whether to make changes. While the three senior doctors were working, they were unable to access all patient-related medical information. Finally, all the images of the two groups of experiments were exported by a doctor.

### Extraction of radiomic features

All radiomics feature were automatically using imaging Biomarker Explorer (IBEX) software (MD Anderson Cancer Center, TX, USA), which is an open infrastructure software platform that flexibly supports common radiomics workflow tasks such as multimodality image data import and review, development of feature extraction algorithms, model validation, and consistent data sharing among multiple institutions [26]. In this study, we named the features with different names under the same matrix as parent features, and the features with the same name extracted from different angles and different distances as child features, in other words, we did not average the feature values of the same name from the same matrix extracted from different angles and distances.

**Fig. 2:**
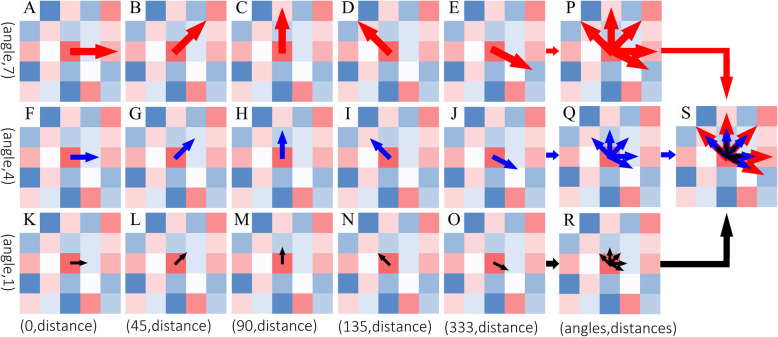
Algorithm rules of feature extraction. Direction and length of the arrow represent the angle and distance of feature extraction, respectively. Child feature(**A** ~ **O**); Parent feature(**P** ~ **S**)

This process had been shown in Fig. 2.

### Statistical analysis

The Intra-class correlation coefficient (ICC) was used to evaluate reproducibility [27], ICC ranges from 0 to 1, where 0 indicates no repeatability and 1 indicates full repeatability. Intraclass correlation coefficients were defined as poor (ICC < 0.4), fair (0.4 ≤ ICC < 0.6), good (0.6 ≤ ICC < 0.75), and excellent (ICC ≥ 0.75) as reported previously [28]. Only the feature with excellent repeatability will be included in the follow-up analysis. The Mann-Whitney U test [29] was performed to analyze the differences between PIPT and peripheral lung cancer, this process of statistical analysis was implemented on Matlab (version 2014b, www.mathworks.com). Radiomics features that can distinguish between peripheral lung cancer and PIPT were obtained using logistic regression analysis on Medcalc (version 18.2.1, http://www.medcalc.org). The diagnostic performance of the radiomic features was illustrated by the receiver operating characteristic (ROC) curve with indices of the area under the curve (AUC), confidence Interval, sensitivity and specificity, and compared by the Delong test. The criterion value distinguished peripheral lung cancer and PIPT was determined by the maximum of the Youden index [30] (calculating the sum of specificity and sensitivity at all possible thresholds, then subtracting one), *P*-value was corrected by false discovery rate (FDR) to adjust for multiple comparisons [31], *P*-value < 0.05 was considered statistically significant. In addition, we average the feature values of the same name extracted from different angles and distances as new features and observe their diagnostic capabilities.

**Fig. 3:**
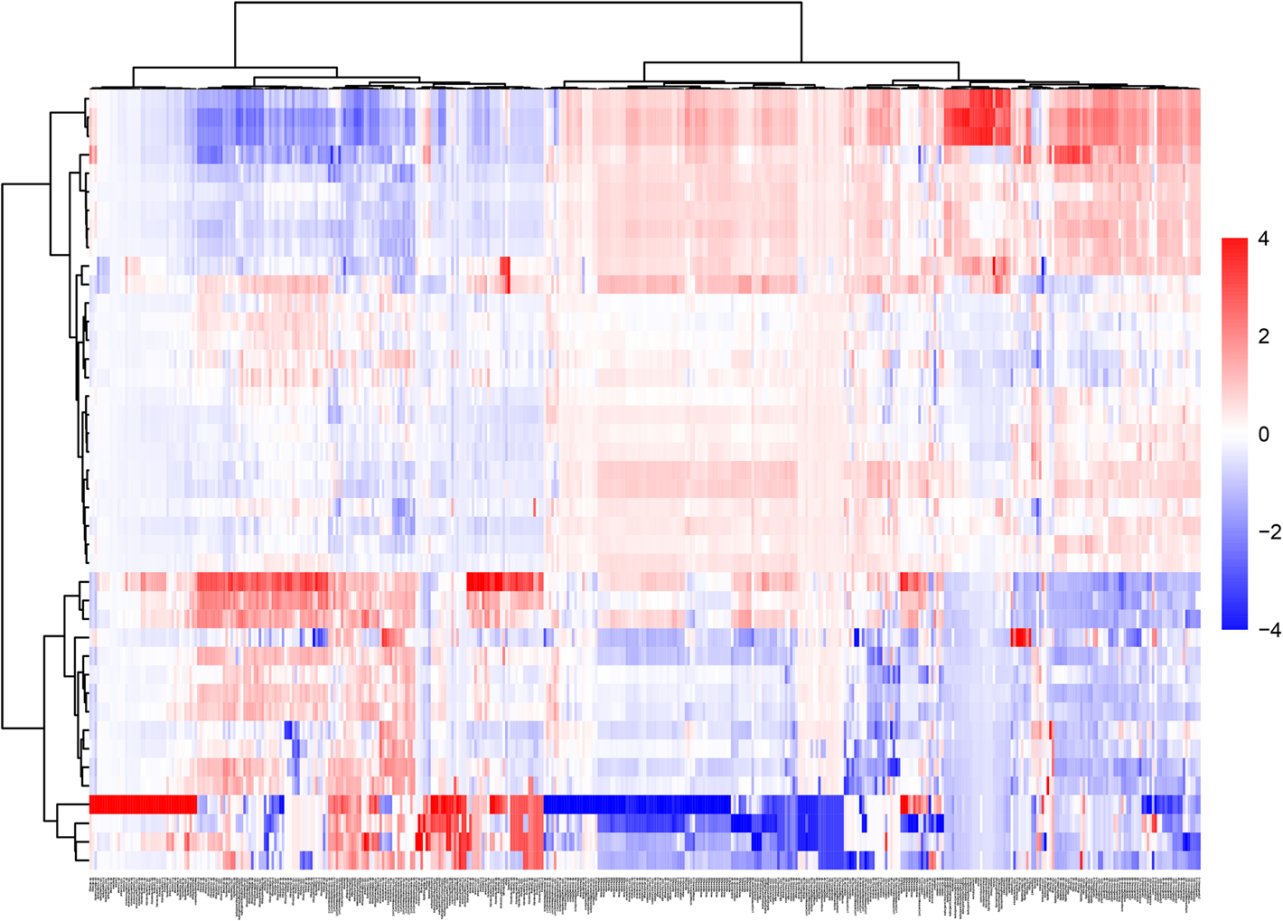
Unsupervised hierarchical clustering heat map of radiomic features extracted from CT images from 42 patients. In the heat map, each row of the heat map represents a patient, and each column represents a radiomic feature extracted from the patient’s CT images. The differences in feature values were visualized using red to represent higher than average and blue to represent lower than average

The data from this study were z-score transformed across each radiomic features and displayed as a heat map with ward. D agglomeration method. Figure 3 represents a heat map of all radiomic feature extracted.

## Results

### Patient characteristics

A total of 21 PIPT patients were enrolled in this study, including 15 males and 6 females (maximum age 74 years, minimum age 44 years, median age 58 years). In the second step, 21 patients with peripheral lung cancer were randomly collected, including 13 males and 8 females (maximum age 78 years, minimum age 44 years, median age 67 years). 14 were diagnosed with adenocarcinoma, 6 with squamous cell carcinoma, and 1 with atypical carcinoid. The clinical characteristics of the patients in this study were shown in Table 1.

**Table 1:** Clinical case information of patients with peripheral lung cancer and PIPT enrolled in this study

Characteristics	peripheral lung cancer	PIPT
**Age**, Mean ± SD (years)	63.62 ± 8.62	57.68 ± 8.61
**Gender**		
Male	15	13
Female	6	8
**Tumor volume** (cm^3^)		
Minimum value	1.65	1.05
Maximum value	64.29	82.08
Mean value	14.62	18.17
**Maximum diameter of tumor** (cm)		
Minimum value	1.61	1.69
Maximum value	6.4	4.97
Mean value	3.30	2.75
**SUV**		
Minimum value	3.6	2.6
Maximum value	26.2	17.3
Mean value	12.73	10.46

### Feature results

In this study, a total of 435 radiomics features were extracted (composed of 368 texture features, 18 shape features, and 49 tumor intensity features), According to the different calculation methods, these features were divided into 5 categories. Among them, the gray-level co-occurrence matrix (GLCM,22 parent features, 330 child features), gray-level run length matrix (GLRLM, 11 parent features, 33 child features), intensity histogram (IH, 9 parent features,49 child features), gray-level neighbor intensity difference matrix (GLNIDM, 5 parent features, none), shape (null,18 patient features, none).

### Statistical results

As shown in Table 2, a total of 361 radiomic features (ICC ≥ 0.75) show good repeatability. Among them, the ability of repeatable shape features is up to 100% (18/18). followed by the features belonging to IH and GLCM reached 85.71% (42/49), 84.24% (278/330), respectively.

**Table 2:** The number of features grouped according to ICC

Matrix	Poor (ICC < 0.4)	Fair (0.4 ≤ ICC < 0.6)	Good (0.6 ≤ ICC < 0.75)	Excellent (0.75 ≤ ICC)	Total
**GLCM**	25	27	72	206	330
**GLRLM**	1	11	7	14	33
**IH**	0	7	25	17	49
**NIDM**	1	2	0	2	5
**SHAPE**	0	0	6	12	18

All statistical differences of PIPT and peripheral lung cancer were tested by the Mann-Whitney U test. A total of 29 feature differences were found to be statistically significant, of which the GLCM has 22 child features, which belong to 5 parent features, respectively, 1 parent feature in IH, 1 parent features in NIDM, 5 parent features in shape, and their child features are all 0. The 30 radiomics features with statistically significant differences are shown in Table 3**.**

**Table 3:** Feature parameters differentiating between pulmonary inflammatory pseudotumor and peripheral lung cancer

Category	Parent Feature	Child Feature	*P*_*1*_ -value	*P*_*2*_ -value
**GLCM**	Cluster Prominence	135–1Cluster Prominence	0.0325	0.2731
	Correlation	333–1 Correlation	**0.0111**	**0.0004**
		333–4 Correlation	**0.0497**	**0.0070**
		45–1 Correlation	**0.0128**	**0.0014**
		45–4 Correlation	**0.0020**	**0.0021**
		45–7 Correlation	**0.0025**	**0.0079**
		90–4 Correlation	**0.0028**	**0.0029**
		90–7 Correlation	**0.0086**	**0.0020**
		135–7 Correlation	**0.0265**	**0.0368**
	Information Measure Corr1	333–4 Information Measure Corr1	0.0157	0.1208
		0–1 Information Measure Corr1	**0.0128**	**0.0384**
		45–1 Information Measure Corr1	**2.97E-5**	**0.0018**
		90–1 Information Measure Corr1	**5.7E-5**	**0.0002**
		90–4 Information Measure Corr1	0.0489	0.1070
		135–1 Information Measure Corr1	**0.0137**	**0.0158**
	Information Measure Corr2	333–1 Information Measure Corr2	**0.0442**	**0.0140**
		333–4 Information Measure Corr2	0.0442	0.0955
		0–1 Information Measure Corr2	0.0497	0.0978
		45–1 Information Measure Corr2	**0.0020**	**0.0184**
		90–1 Information Measure Corr2	**0.0083**	**0.0130**
		135–1 Information Measure Corr2	**0.0168**	**0.0273**
	Inverse Diff Moment Norm	135–7 Inverse Diff moment Norm	0.0391	0.2151
**IH**	Range	None	**0.0169**	**0.0232**
**NIDM**	Complexity	None	0.0268	0.0595
**SHAPE**	Compactness2	None	**0.0017**	**0.0027**
	Roundness	None	**0.0083**	**0.0102**
	Spherical Disproportion	None	**0.0017**	**0.0020**
	Sphericity	None	**0.0017**	**0.0028**
	Surface Area Density	None	**0.0207**	**0.0443**

Note: The significant difference index of Mann-Whitney U test, P1-value; the significant difference index of Binary logistic regression, P2-value; indicates a significant difference (*p* < 0.05)

Binary logistic regression model analysis showed that 23 of the 29 child features were significantly different and could be used to distinguish PIPT from peripheral lung cancer. These 23 child features respectively belong to GLCM (parent feature correlation (*n* = 8), parent feature information measure corr1 (*n* = 4) and parent feature information measure corr2 (*n* = 4))、 IH (parent feature range(*n* = 0)) 、GLNIDM (parent feature texture strength(*n* = 0)) and shape (parent feature compactness2(*n* = 0), Roundness(*n* = 0), parent feature spherical disproportion(*n* = 0), parent feature sphericity (*n* = 0) and parent feature surface area density(*n* = 0)),respectively. (Table 3).

**Fig. 4:**
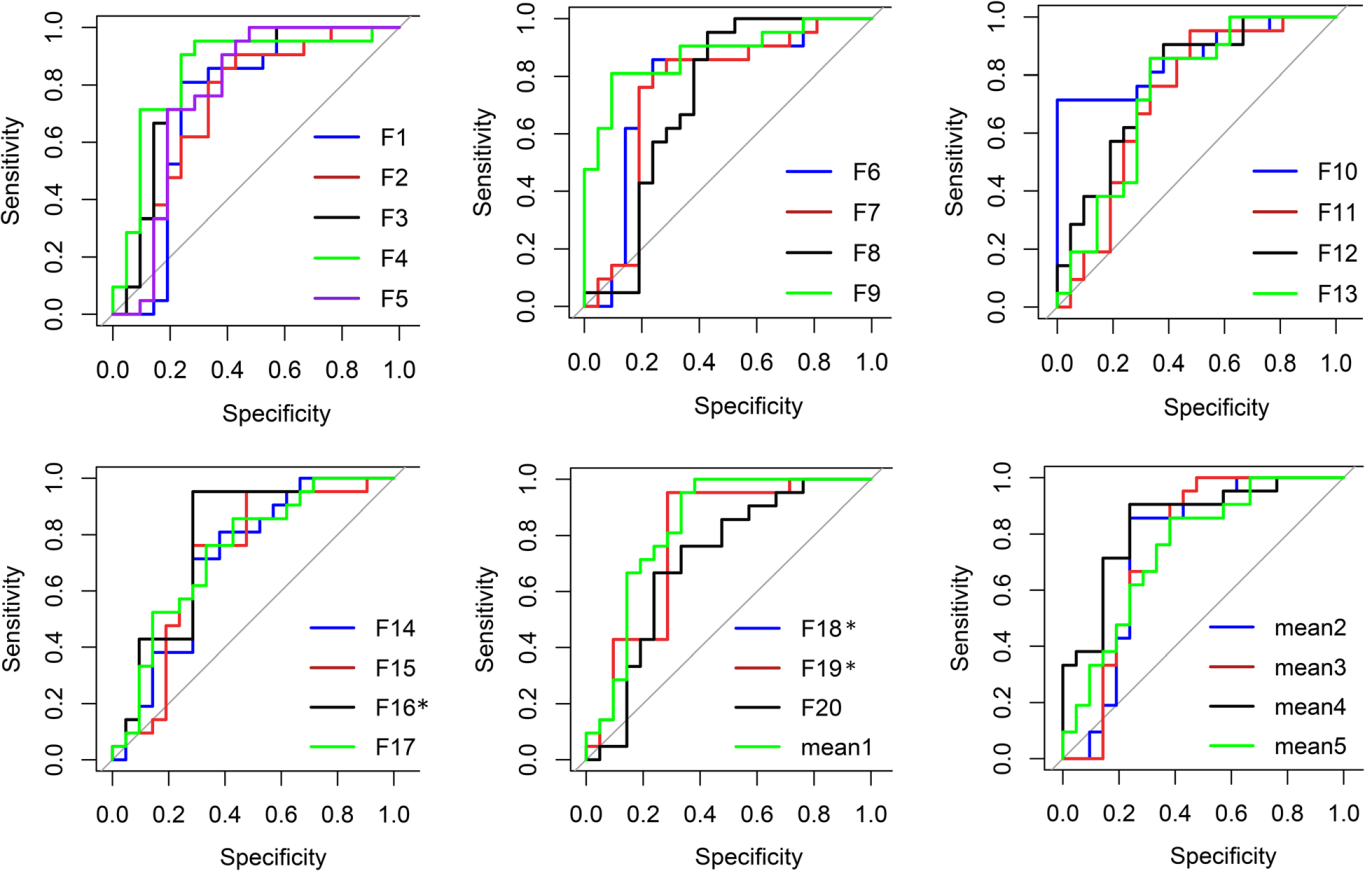
ROC curses of radiomic features. * - Line coincidence

ROC curves of 25 features were performed to evaluate the ability of features differentiating peripheral lung cancer from PIPT. The curves (AUC < 0.7) was abandoned in this study, because of its limited discriminant ability. Finally, A total of 20 ROC curves of features were obtained in this study. In addition, we calculated the average value of the features at the same angle and different distance and drew ROC curves, which were curve Mean1 and Mean2. we also calculated the average value of the features at different angles and different distances and the drew of ROC curves were curve mean3, mean4, mean5, respectively. The *P*-values of statistical differences among ROC were 0.0499 (F9, F20), 0.0472 (F10, F11), and 0.0145 (F11, Mean4), and the others were 0.5908 ± 0.2803. All ROC curves are shown in Fig. 4.

At the same time, we calculate the AUC, sensitivity, specificity, cut-off value, and Youden index. We calculated their interquartile range (IQR) and median values for features with discriminating ability, respectively. The specific values of all statistical parameters differentiating between peripheral lung cancer and PIPT are shown in Table.4.

**Table 4:** Statistical parameters differentiating between peripheral lung cancer and PIPT

Feature	Peripheral Lung Cancer		PIPT		AUC	Confidence Interval (%)	Sensitivity(%)	Specificity(%)	Cutoff-Value	Youden-Index
	IQR	median	IQR	median						
F1	0.0429	0.7334	0.0874	0.7913	0.730	55.74 ~ 90.29	80.95	76.19	0.7657	0.5714
F2	0.1056	0.6783	0.1218	0.7846	0.726	56.02 ~ 89.11	80.95	66.67	0.7401	0.4762
F3	0.1399	0.0738	0.1480	0.2719	0.800	69.04 ~ 96.94	90.00	70.59	0.2047	0.6059
F4	0.1889	−0.0284	0.2054	0.1434	0.806	71.76 ~ 97.85	88.89	68.75	0.0246	0.5764
F5	0.1965	0.2102	0.1405	0.3946	0.774	61.64 ~ 0.9306	90.48	65.00	0.3589	0.5548
F6	0.1370	0.0223	0.1438	0.1954	0.754	61.65 ~ 93.45	84.21	72.22	0.1234	0.5643
F7	0.1467	−0.0576	0.2119	0.410	0.718	58.29 ~ 91.37	73.68	76.47	−0.0316	0.5015
Mean1	0.1500	0.2280	0.1403	0.4112	0.800	77.01 ~ 98.50	95.24	71.43	0.3512	0.6667
Mean2	0.1647	0.1211	0.1504	0.2990	0.753	58.75 ~ 91.82	85.71	76.19	0.1855	0.6190
Mean3	0.1372	0.2362	0.1453	0.3776	0.751	58.65 ~ 91.46	90.48	61.90	0.3055	0.5238
F8	0.0436	−0.3821	0.1112	−0.4560	0.726	55.63 ~ 89.49	95.24	57.14	−0.4461	0.5238
F9	0.0409	−0.2944	0.1154	0.3496	0.878	77.01 ~ 98.50	80.95	90.48	−0.3106	0.7143
F10	0.0506	−0.3605	0.0892	−0.4266	0.864	75.06 ~ 97.73	71.43	100.00	−0.3813	0.7143
F11	0.0389	−0.2849	0.1490	−0.3602	0.723	55.99 ~ 88.68	95.24	52,38	−0.3602	0.4762
Mean4	0.0329	−0.3328	0.0751	−0.3978	0.841	71.78 ~ 96.48	90.48	76.19	−0.3624	0.6667
F12	0.0428	0.8997	0.0692	0.9302	0.780	63.75 ~ 92.26	85.71	66.67	0.9175	0.5238
F13	0.0190	0.9330	0.0450	0.9623	0.739	58.18 ~ 89.66	85.71	66.71	0.9567	0.5238
F14	0.0378	0.8930	0.0788	0.9202	0.717	55.49 ~ 87.82	71.43	71.43	0.8975	0.4286
Mean5	0.0244	0.9065	0.0625	0.9419	0.751	60.00 ~ 90.12	85.71	61.91	0.9252	0.4762
F15	115	346	189	494	0.717	54.55 ~ 88.36	76.19	71.43	400.0000	0.4762
F16	0.1460	0.5928	0.4127	0.2817	0.785	63.38 ~ 93.54	95.24	71.43	0.3259	0.6667
F17	0.1063	0.3569	0.2001	0.2136	0.739	58.48 ~ 89.37	85.71	57.14	0.2454	0.4286
F18	0.0953	1.1904	0.5439	1.5253	0.785	63.38 ~ 93.54	95.24	71.43	1.3783	0.6667
F10	0.0680	0.8400	0.2671	0.6556	0.785	63.38 ~ 93.54	95.24	71.43	0.6882	0.6667
F20	1.7698	2.9145	5.3141	4.5316	0.710	54.38 ~ 87.57	66.67	76.19	2.9718	0.4286

Note: F1-GLCM-333-1-correlation, F2-CLCM-45-1-correlation, F4-GLCM-45-4-corr-elation, F6-GLCM-45-7 correlation, F6-GLCM-90-7-correlation, F7-CLCM-135-7-correlation, F8-GLCM-0-1-information-measure corr1, F9-GLCM-45-1-information measure corr1, F10-GLCM-90–1-information measure corr1, F11-GLC-M-135-1-information measure corr1, F12-GLCM-45-1-information measure corr2, F13-IGLCM-information measure-90–1-information corr2, F14-GLCM-135-1-information corr2, F15-IH-range, F16-shape-compactness2, F17-shape-roundness, F19-shape-sphericity, F20-shape-surface area density, mean1-mean(F2+ … … + F4), mean2-mean(F5+ … … F6), mean3-mean(F1+ … … F7), mean4-mean(F8+ … … F11), mean5-mean(F12+ … … + F14)

## Discussion

The results of this study indicated that there was a statistically significant difference between the radiomic features extracted from patients with peripheral lung cancer and those extracted from patients with PIPT. We assumed that this statistically significant difference between peripheral lung cancer and PIPT in this study might be related to the pathological microstructure differences between the two tissue types. Adenocarcinoma may consist of some cubic tumor cells and some columnar [32]. The pathology of primary lung adenocarcinoma is mostly papillary type, others are solid, lepidic, acinar, and micropapillary subtypes., which is often diagnosed as a mixture of multiple subtypes [33]. The pathological subtypes of lung squamous cell carcinoma include small cell, clear cell, basaloid and papillary subtypes [6, 34], in which papillary squamous cell carcinoma is usually characterized by exogenous endobronchial growth [35]. The histological types of small cell carcinoma are oat, intermediate and mixed subtype [6]. On pathological smears, most of the cancer cells are quasi-round or fusiform, with few cytoplasm and naked nuclei, which are very similar to lymphoma cells, and there are often mixed non-nucleated necrotic cells or extensive necrotic areas [6]. Large cell cancer cells often show sheets and nests distribution. This kind of cancer cells has rich cytoplasm, vesicular nuclei and prominent nucleoli [6, 36]. The histological structure of PIPT is complex and often accompanied by a mixture of different numbers of cells. The main cell types of PIPT are spindle cells (myofibroblasts and fibroblasts) and chronic inflammatory cells (especially plasma cells, lymphocytes and macrophages) [17, 37, 38]. The histology of PIPT can be divided into three types: (1) fibrous histiocytic type characterized by fusiform myofibroblasts and most common (2) under the microscope, most of the patients with organized pneumonia are foam cells, eosinophils and multinucleated cells (3) Lymphocyte type is dominated by lymphocytes and plasma cells, which is the most rare type [18, 39, 40].

The slight difference in pathology between peripheral lung cancer and pulmonary inflammatory pseudotumor may be reflected in the attenuation of CT. Correlation is a value between 0 (uncorrelated) and 1 (perfectly correlated) showing the linear dependency of gray level values to their respective voxels in the GLCM [41], Information Measure Corr1 and Information Measure Corr2 are two features that assessing the correlation between the probability distributions of two voxels (quantifying the complexity of the texture) [41, 42]. The median values of Correlation(F1- Mean3)、Information Measure Corr1(F8-Mean4) and Information Measure Corr2(F12-Mean5) of PIPT in this study are mostly higher than that of peripheral lung cancer, which may be related to the distribution of inflammatory cells in PIPT. The specific difference of different radiomic features were described in Table 2. Relevant reports show that myofibroblasts and histiocytes play a dominant role in PIPT and are arranged in spirals or flakes [18, 39]. This may be because PIPT necrosis is relatively less than lung cancer [9, 43, 44], Lung cancer tissue grows rapidly and is prone to focal necrosis, which may lead to the larger values of Range(F17) and Texture Strength(F18) of PIPT compared with that of lung cancer. In this study, the shape features Compactness2, Spherical Disproportion and Spherical are the quantification of tumor roundness, and there is a certain correlation between these three features in definition. In this study, the ROC curves of these three features(F16, F18, F19) coincide, which also confirms this fact. This may be that the most of the peripheral lung cancer is spherical, and the most of the PIPT are found in the lower lobe of the lung and are round or oval [17, 20]. Although the morphological features of peripheral lung cancer and pulmonary inflammatory pseudotumor are highly similar, the probability of occurrence of the same imaging features may be different in the cohort of patients [39, 45]. this may be an important reason why most of the differences between peripheral lung cancer and PIPT in this study belong to shape features.

In addition, in this study, we found that the features extracted from different angles and distances had no special advantage in distinguishing peripheral lung cancer from PIPT. On the contrary, the features extracted from different angles and different distances have relatively high discriminating ability, such as 45–7 Correlation (F4), 45–1 Information Measure Corr1 (F9), 90–1 Information Measure Corr1 (F10). This may be related to the directionality of the microstructure of the lung in space.

Using texture features to distinguish benign and malignant tissues has been reported in many articles. Tsai et al. [46] reported that Texture features can be used to distinguish nasopharyngeal carcinoma from normal nasopharyngeal tissue, and the statistical difference in texture features between nasopharyngeal carcinoma and normal nasopharyngeal tissue may be related to the loss of stripe structure in normal nasopharyngeal tissue. and this finding had been confirmed on MRI images. Alilou et al. [47] had shown that lung adenocarcinoma and granuloma can be diagnosed by shape-based features (sphericity and roughness), and the two lesions were difficult to distinguish in pathology. These reports give great inspiration to the development of our study.

The reproducibility of radiomic features has long been an inescapable topic in the field of radiomics research. Variations in patient location, feature extraction software, imaging device and segmentation will have an unpredictable impact on the repeatability of radiomic features. If features with relatively low robustness are used in the study, they may perform poorly when tested by new data sets and even seriously affect our conclusions. The results of this study showed that there were differences in the repeatability of features extracted from ROI segmented by manual. As manual segmentation was easily affected by different levels of observers, we used ICC to filter features with high robustness, but this still can not meet our needs. In the follow-up research, a variety of image segmentation methods will be used to screen highly robust features and verify our conclusions, such as automatic and semi-automatic segmentation methods.

There are many limitations to our research. First of all, the number of patients is small, due to the relatively low incidence of PIPT, to a certain extent, it limits our collection of relevant medical images. In addition, this study lacks directly related pathological experimental tissue specimens, so that we can not directly confirm our findings, and the hypothesis of related problems can only be based on previous reports. Finally, all the medical images in this study are obtained from the same PET/CT model. Further studies are needed to determine whether the images obtained from different PET/CT machines can get the same conclusion. In the following research, we will enlarge the sample size as much as possible and apply machine learning and deep learning methods to verify our conclusions.

## Conclusions

There were significant differences in radiological characteristics between peripheral lung cancer and PIPT, among which Information Measure Corr1 features (F11, F12) showed the highest ability to distinguish peripheral lung cancer from PIPT.

## Data Availability

The datasets used and analyzed during the current study available from the corresponding author on reasonable request (yinyongsd@126.com).

## Abbreviations

18F-FDG PET/CT: 18F-fluorodeoxyglucose positron emission tomography/computed tomography
PIPT: Pulmonary inflammatory pseudotumor
ROI: Regions of interest
ICC: Intra-class correlation coefficient
ROC: Receiver operating characteristic
AUC: Area under the curve
GLRLM: Gray-Level Run Length Matrix
GLCM: Gray-Level Co-occurrence Matrix
IH: Intensity Histogram
GLNIDM: Gray-level Neighbor Intensity Difference Matrix
IQR: Indicates interquartile range
FDR: False discovery rate
